# Supplementary material for: Reactive Molecular Dynamics Simulations of Polystyrene Pyrolysis
Source: Int J Mol Sci. 2023 Nov 16;24(22):16403. doi: 10.3390/ijms242216403 (PMC10671678; doi:10.3390/ijms242216403)
Supplement: Supplementary file 1 [file ijms-24-16403-s001.zip › ijms-2678689-supplementary.pdf]

# Supporting Information

## Reactive Molecular Dynamics Simulations of Polystyrene Pyrolysis

Chao Li[1], Zhaoying Yang[1], Xinge Wu[1], Shuai Shao[1], Xiangying Meng[1, 3]* and Gaowu Qin[2, 3]

[1]*College of Sciences, Northeastern University, Shenyang 110819, China*

[2] *Key Laboratory for Anisotropy and Texture of Materials (MoE), School of Materials Science and Engineering, Northeastern University, Shenyang 110819, China*

[3]*Institute of Materials Intelligent Technology, Liaoning Academy of Materials, Shenyang 110004, China*

*Corresponding author. Email: x_y_meng@.mail.neu.edu.cn (Xiangying Meng)

# 1. Thermodynamic parameters during pyrolysis simulations

To evaluate the pressure under the NVT ensembles, we plot pressure change with time at various constant temperature simulations in Figure S1. Based on the carbon phase diagram, we know that the coordination number of carbon is 3 (graphite structure) at low pressure, and carbon changes to a diamond structure at 5 Gpa pressure. In our simulations, the pressure is below the phase transition points, so the pressure will not induce intrinsic changes to the microstructure but may affect the parameters, such as bond length, etc. In addition, as we list in the references, many molecular dynamics studies on the pyrolysis of organic matter have used NVT systematic simulations, obtaining better results in line with experiments.

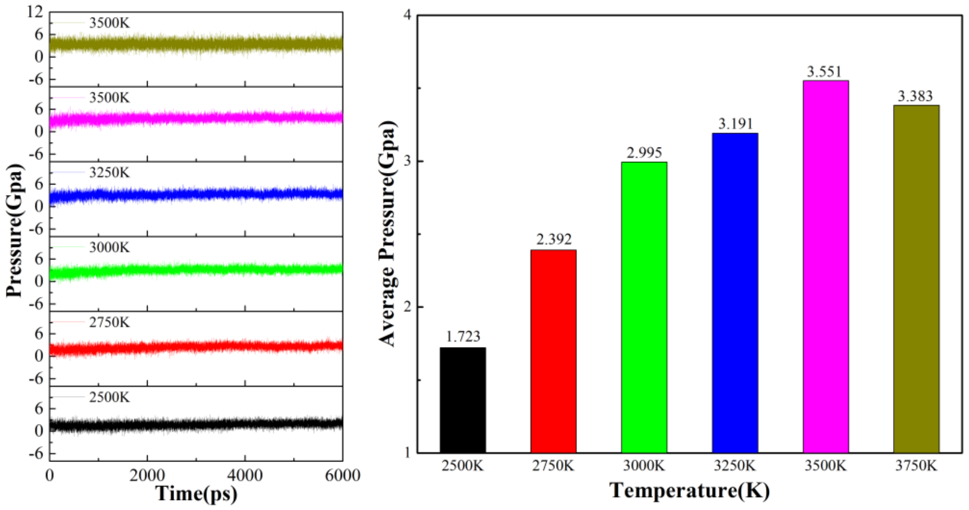


(a) (b)

Figure S1(a). Variation of pressure with time during thermostatic simulation;

(b). Comparison of mean pressures during different thermostatic simulations.

The changes in temperature, total energy, kinetic energy, potential energy, and bond energy reflect the breaking and formation of chemical bonds during the simulation process [1-4]. For the simulated system warmed to different target temperatures at a warming rate of 50 K/ps, the total and potential energies of the structure obtained at the warming to different temperatures are shown in Figure S2 (a): the total energy and potential energy of the obtained structures increase with the rate of warming up. This further indicates that the excessively high temperature accelerates the pyrolysis of the molecular chains in the system. The variation of temperature and potential energy with time during the constant temperature simulation is shown in Figure S2 (b). It can be seen that the groups show a transient increase in potential energy within the system at the early stage of constant temperature simulation. The reason for the increase in potential energy at this time is that the structure in the system is undergoing sufficient pyrolysis and absorbing energy. As the reaction proceeds, the potential energy of each group also decreases gradually before 2500 ps. However, the rate of decrease is clearly proportional to the temperature. The peak energy during the isothermal simulation shows a significant span, and the peak occurs earlier with the increase in the temperature of the simulation. The structure at the peak energy in these groups also includes a carbon-hydrogen macromolecule structure and some hydrogen molecules. Only the amount of hydrogen in these groups varies considerably. The influence of temperature on the molecular structure and energy variation within the system is more pronounced.

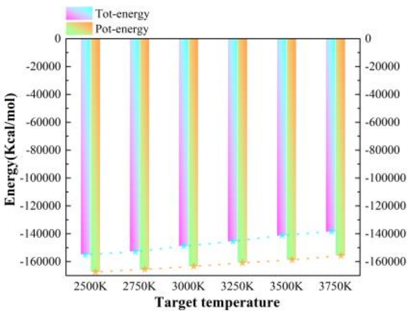


(a)

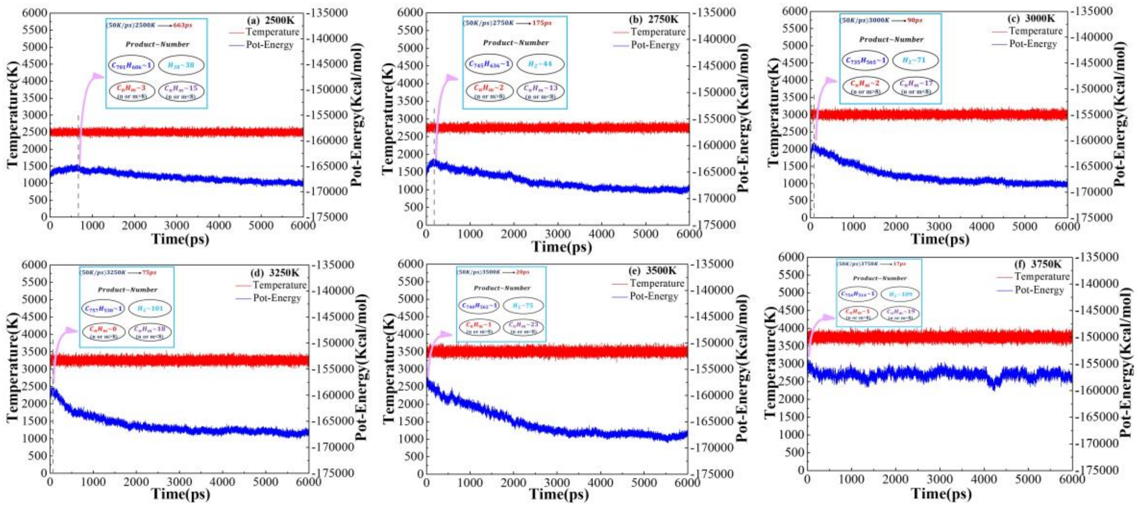


(b)

Figure S2. Changes in temperature and energy

(a) At 50K/ps ramping rate to different target temperatures

(b) Thermostatic simulation of the 6ns process

## 2. The detailed hydrogen evolution during pyrolysis

The simulated pyrolysis of polystyrene produces small molecule products of pyrolysis, mainly styrene and styrene compounds, before the apparent precipitation of hydrogen, which is consistent with the conclusions obtained in the experiments [5]. From the above analysis, we can find that the energy change of the pyrolysis reaction is closely related to the change in the quantity of various intermediate products. During the constant temperature simulation process, the simulated system continues to be cleaved to generate many kinds of hydrocarbon small molecules, mainly in the first 2500ps, at which time the system as a whole is exothermic. After 2500 ps, the system does not undergo intense pyrolysis under different simulation conditions. The dehydrogenation reaction dominates, and hydrogen is also the main product of the simulated process. Therefore, it becomes crucial to track and analyze the rate of hydrogen production throughout the reaction.

*2.1 Formation of the dominant product (hydrogen)*

Further analysis of the change in the number of hydrogen molecules produced by each group with respect to the time of the thermostatic simulation reveals that the rate at which the system removes H atoms in the form of hydrogen during the thermostatic simulation is also limited by the temperature. From Figure S3 (a), we can find that the hydrogen production rate due to the same temperature is almost the same for all groups of constant temperature simulations after warming up to 3000 K either

by low or high rate. Figure S3 (b) shows that the higher the temperature, the better the removal of hydrogen atoms, except for the UHTs group. In contrast, the changes in the amount of precipitated hydrogen were similar in the PTs and HTs groups. Furthermore, the trends of the number of hydrogen with simulation duration for each group and the total number of molecules with simulation duration during thermostatic simulation (see Figure S4 for details) have a synchronous nature. This further indicates that the molecules in the system during the constant temperature simulation remove H atoms in the form of hydrogen while generating macromolecular structures. Hydrogen gas generation is mainly concentrated in the earlier part of the thermostatic simulation.

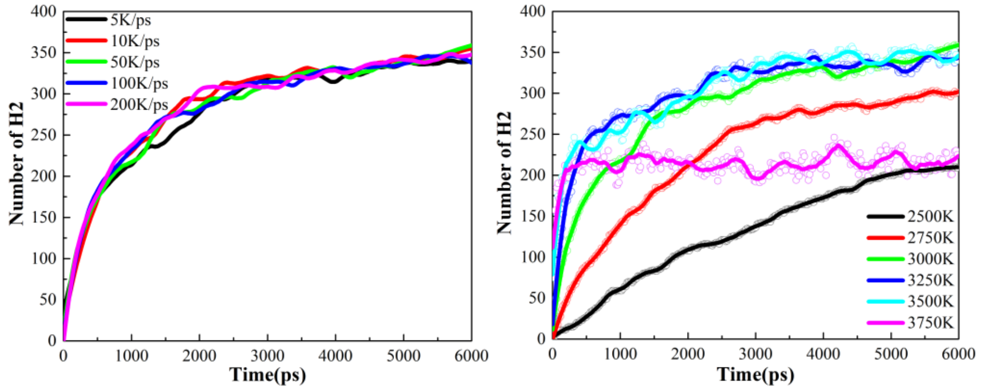


(a) (b)

Figure S3. Variation of hydrogen quantity with time during the simulation at constant temperature

(a)Simulation of constant temperature after warming to 3000 K at different warming rates;

(b) Simulation of constant temperature after warming up to different target temperatures with a 50K/ps ramp rate.

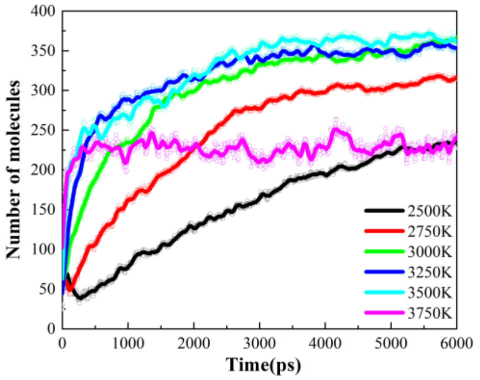


Figure S4. Variation of the total number of molecules with time at different thermostatic simulation temperatures.

*2.2 Hydrogen content in the carbon network structure within the system*

After 6 ns of constant-temperature simulation, while the loss of hydrogen decreases the H/C values of the most significant molecular weight carbon clusters at different temperatures, the amount of H2 produced increases. The removal of hydrogen atoms in the groups with too low (2500K) or too high (3750K) temperatures seems to have reached a "bottleneck". The remaining groups still have a small amount of hydrogen attached to the benzene ring structure, mainly in a single or two adjacent states in the benzene ring and less in the form of multiple hydrogen adjacent states. This group at 3500 K has the lowest carbon and hydrogen content in the final macromolecular structure compared to the rest of the groups. The carbon and hydrogen contents and H/C values of the final macromolecular of each group are shown in Table 1 below:

**Table 1.** The H/C value, carbon content, and hydrogen content of the maximum molecular structure in the final system of each group.

| Group | Carbon content | Hydrogen content | H/C value |
|---|---|---|---|
| 5K/ps (3000K) | 96.6% | 7% | 0.074 |
| 10K/ps (3000K) | 99.3% | 9.6% | 0.099 |
| 50K/ps (3000K) | 98.3% | 8.3% | 0.085 |
| 100K/ps (3000K) | 97.1% | 10.1% | 0.106 |
| 200K/ps (3000K) | 98.6% | 10.2% | 0.105 |
| 50K/ps (2500K) | 94.5% | 35.4% | 0.379 |
| 50K/ps (2750K) | 97.4% | 19.3% | 0.200 |
| 50K/ps (3250K) | 96.8% | 10% | 0.105 |
| 50K/ps (3500K) | 93.4% | 6.4% | 0.070 |
| 50K/ps (3750K) | 96.3% | 41.1% | 0.432 |

*2.3 Direction of Hydrogen Aggregation*

We find two groups that have obvious directions of hydrogen gas precipitation along the coordinate axes when calculating the density distribution of hydrogen atoms using the OVITO software. Firstly, in the group that is heated up to 3000K at 50K/ps, hydrogen gas gradually precipitates from the center towards both sides of the X-axis during the constant temperature simulation. Secondly, hydrogen gas precipitates along one side of the Y-axis as the simulation progresses in the group that is heated up to 3500K at 50K/ps. In the remaining groups, hydrogen gas is distributed almost uniformly throughout the system. The details refer to Figure S5 as follows.

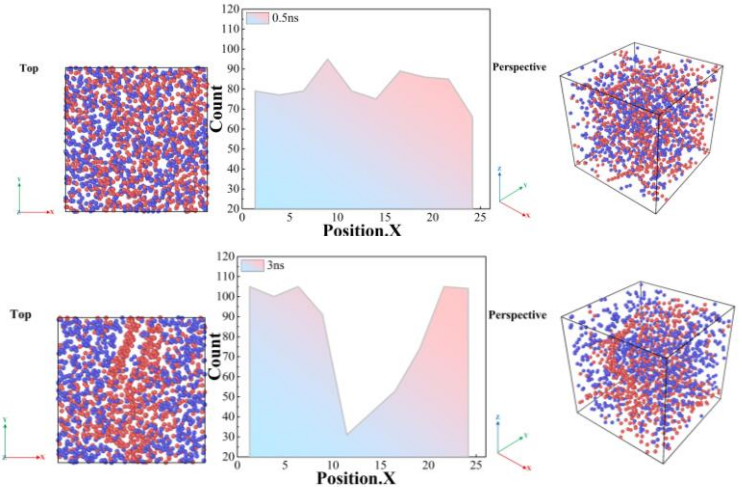

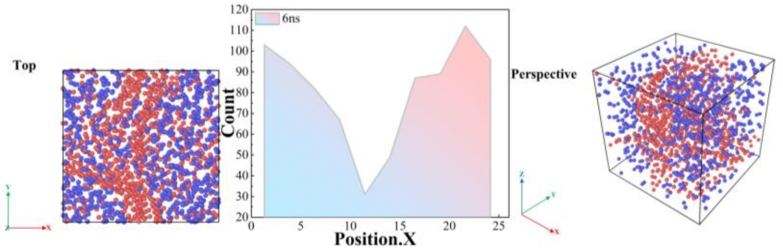


(a)

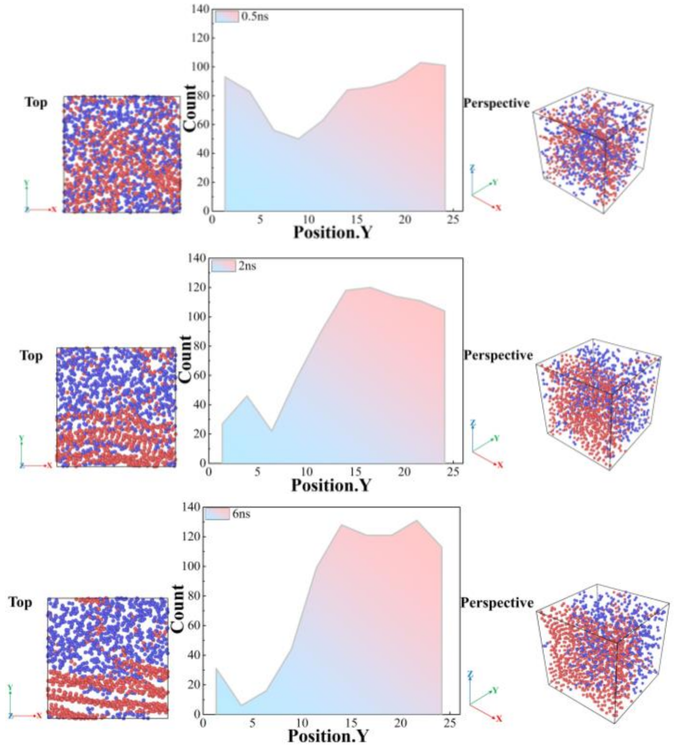


(b)

Figure S5. Hydrogen atom density distribution during constant temperature simulation

(a) ramping up to 3000 K at 50 K/ps

(b) ramping up to 3500 K at 50 K/ps

●-C atom ●-H atom

It can be seen that the groups of PTs and HTs ensure both the removal efficiency of hydrogen atoms and the generation of a more pronounced graphite layer structure. There are some reasons why the final amount of hydrogen precipitated at 2500 K and 3750 K is less, which leads to the incomplete removal of impurity hydrogen atoms. Firstly, limited by the time scale issue in all molecular dynamics methods, our simulation time frame is relatively short because we use high temperatures to accelerate the chemical reaction. In contrast, at lower temperatures (e.g., at 2500 K), the simulation time would be very long to produce more hydrogen. Second, pyrolysis experiments on polystyrene are usually performed under continuous gas flow, and the pyrolysis products are carried away with the gas flow, making it challenging to observe large swaths of carbon network structure. Whereas we perform the simulation in the NVT integration, the whole simulation system is closed relative to the experiment. So too high temperatures (e.g., at 3750K) are likely to be due to secondary solid reactions of internal molecular products caused by artificially elevated high temperatures in the closed simulation

environment. This makes the removal of hydrogen less ideal. This also leads to the difficulty of observing the crosslinked carbonization of polystyrene in experiments, whereas the process of dehydrogenation of polystyrene carbonization can be better observed in simulations. As hydrogen atoms are eliminated in the form of hydrogen, the carbon content of the structure gradually increases, eventually forming a stable, randomly folded carbon sheet with high molecular weight.

## 3. The detailed evolution of pore distribution

After reaching the target temperature, the products formed by thermal transformation are relatively diverse as pyrolysis proceeds. Figure S6 shows the changes in the pore size range of each group during the constant temperature simulation process. Different warming rates have little effect on the final pore size distribution of each group of simulated systems at the target temperature of 3000K. For each group at different temperatures, the aperture size inside the system changes drastically in the early stage of the thermostatic simulation. However, in the middle and late stages of the simulation, the aperture size of the group at 2500 K tends to increase, which is related to the instability of the system structure caused by the insufficient simulation time under LTs. The aperture size distribution of the groups under PTs is basically stable. The aperture size of the group at 3500 K also tends to increase, which is related to the emergence of layered graphite structures under HTs. The system structure of the group under UHTs is relatively stable due to the absence of graphitized tissue.

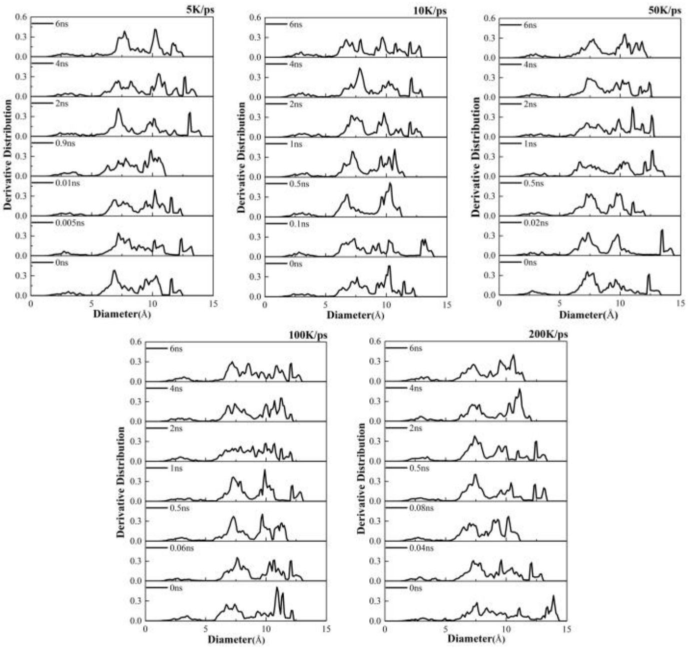


(a)

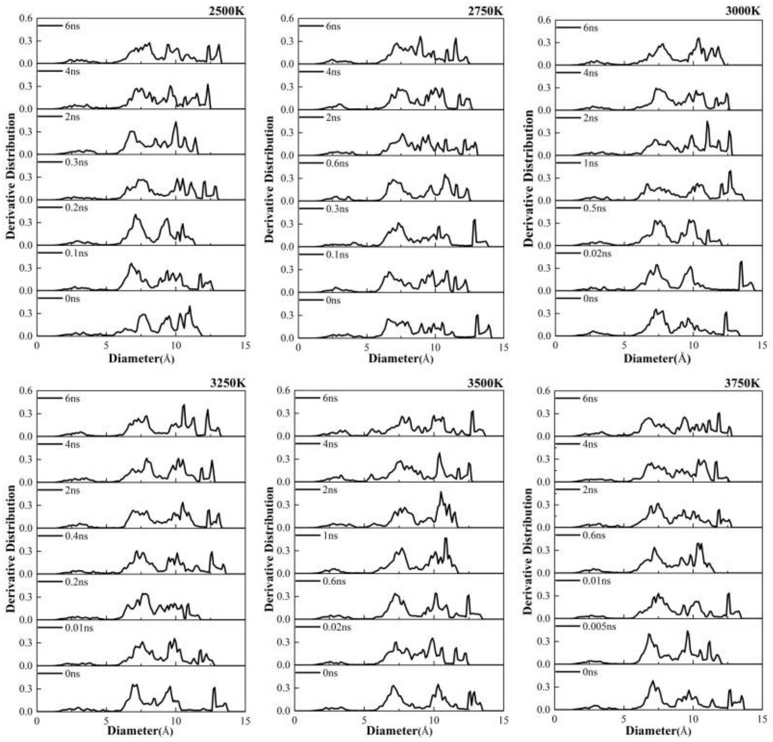


(b)

Figure S6. Pore size distribution of the structure during simulation at constant temperature. The diameter of the probe is 0.5 Å

(a) warm to the same temperature at different warming rates

(b) temperature rise to different temperatures at the same rate of increase

# REFERENCES


[1] Diao, Zh.; Zhao, Y.; Chen, B.; Duan, Ch.; Song, S. ReaxFF reactive force field for molecular dynamics simulations of epoxy resin thermal decomposition with model compound. *Journal of Analytical and Applied Pyrolysis* **2013**, 104, 618-624

[2] Liu, X.; Li, X.; Liu, J.; Wang, Z.; Kong, B.; Gong, X.; Yang, X.; Lin, W.; Guo, L. Study of high density polyethylene (HDPE) pyrolysis with reactive molecular dynamics. *Polymer Degradation and Stability* **2014**, 104, 62-70

[3] Desai, T.G.; Lawson, J.W.; Keblinski, P. Modeling initial stage of phenolic pyrolysis: Graphitic precursor formation and interfacial effects. *Polymer* **2011**, 52, 577-585

[4] Zheng, M.; Li, X.; Liu, J.; Wang, Z.; Gong, X.; Guo, L.; Song, W. Pyrolysis of Liulin Coal Simulated by GPU-Based ReaxFF MD with Cheminformatics Analysis. *Energy & Fuels* **2014**, 28, 522-534

[5] Onwudili, J.A.; Insura, N.; Williams, P.T. Composition of products from the pyrolysis of polyethylene and polystyrene in a closed batch reactor: Effects of temperature and residence time. *Journal of Analytical and Applied Pyrolysis* **2009**, 86, 293-303
